# *Salvia sclarea* L. Essential Oil Extract and Its Antioxidative Phytochemical Sclareol Inhibit Oxytocin-Induced Uterine Hypercontraction Dysmenorrhea Model by Inhibiting the Ca^2+^–MLCK–MLC20 Signaling Cascade: An Ex Vivo and In Vivo Study

**DOI:** 10.3390/antiox9100991

**Published:** 2020-10-14

**Authors:** Jennifer Wong, Yi-Fen Chiang, Yin-Hwa Shih, Chun-Hui Chiu, Hsin-Yuan Chen, Tzong-Ming Shieh, Kai-Lee Wang, Tsui-Chin Huang, Yong-Han Hong, Shih-Min Hsia

**Affiliations:** 1 School of Nutrition and Health Sciences, College of Nutrition, Taipei Medical University, Taipei 11031, Taiwan; jenniferwong21@hotmail.com (J.W.); yvonne840828@gmail.com (Y.-F.C.); hsin246@gmail.com (H.-Y.C.); 2 Department of Healthcare Administration, Asia University, Taichung 41354, Taiwan; s875008@gmail.com; 3 Graduate Institute of Health Industry and Technology, Research Center for Food and Cosmetic Safety, College of Human Ecology, Chang Gung University of Science and Technology, Taoyuan City 33303, Taiwan; chchiu@mail.cgust.edu.tw; 4 Department of Traditional Chinese Medicine, Chang Gung Memorial Hospital, Keelung City 20401, Taiwan; 5 School of Dentistry, College of Dentistry, China Medical University, Taichung 40402, Taiwan; tmshieh@mail.cmu.edu.tw; 6 Department of Dental Hygiene, College of Health Care, China Medical University, Taichung 40402, Taiwan; 7 Department of Nursing, Ching Kuo Institute of Management and Health, Keelung City 20301, Taiwan; kellywang@tmu.edu.tw; 8 Graduate Institute of Cancer Biology and Drug Discovery, College of Medical Science and Technology, Taipei Medical University, Taipei 11031, Taiwan; tsuichin@tmu.edu.tw; 9 Department of Nutrition, I-Shou University, Kaohsiung City 82445, Taiwan; yonghan@isu.edu.tw; 10 Graduate Institute of Metabolism and Obesity Sciences, College of Nutrition, Taipei Medical University, Taipei 11031, Taiwan; 11 School of Food Safety, College of Nutrition, Taipei Medical University, Taipei 11031, Taiwan; 12 Nutrition Research Center, Taipei Medical University Hospital, Taipei 11031, Taiwan

**Keywords:** sclareol, *Salvia sclarea* essential oil, dysmenorrhea, uterine contraction, writhing test

## Abstract

*Salvia sclarea* essential oil is used as an aromatic therapy for dysmenorrhea. Sclareol—one of the natural products isolated from *S. sclarea*—displays anti-inflammatory and antioxidant activities; however, researchers have not yet evaluated the mechanism related to the pain-relieving effect of sclareol. In the present study, we aimed to investigate the potential effect of sclareol in ex vivo and in vivo dysmenorrhea models, as well as its possible mechanism. In the ex vivo study of uterine tissue from Sprague Dawley (SD) rats, the uterine contraction amplitude was observed and recorded. In the in vivo study, we measured the uterine contraction pressure of SD rats and performed writhing tests on mice. The uterine tissues from the writhing test subjects were collected and analyzed by Western blot. The results demonstrated that sclareol inhibited prostaglandin (PG) F_2α_-, oxytocin-, acetylcholine-, carbachol-, KCl-, and Bay K 8644-induced uterine contraction and possessed an analgesic effect in the writhing test. Sclareol affects the Ca^2+^ level and regulates oxytocin receptor (OTR), myosin light chain kinase (MLCK), extracellular signal-regulated kinase, p-p38, cyclooxygenase-2 (COX-2), and phospho-myosin light chain 20 (p-MLC20) protein expression. Integrating these results, we suggest that sclareol is a potential alternative supplement for dysmenorrhea.

## 1. Introduction

Dysmenorrhea is the most common gynecological disease affecting reproductive women globally, with a 16–91% prevalence [1,2]. Instances of dysmenorrhea, leading to absence from school or work, affect performance, as well as causing economic losses [3]. Dysmenorrhea refers to when women undergo lower abdominal pain, who may present with associated symptoms, such as headache, diarrhea, and vomiting [1,4]. However, the reason for dysmenorrhea is still unclear, and most theories have been directed toward the high level of prostaglandin (PG) F2α in the menstrual phase [5]. PG is produced by cyclooxygenase-2 (COX-2) and it can stimulate vasoconstriction of the uterus, leading to ischemia [6]. PG can also cause sensitization of afferent nerves, resulting in the painful symptoms of dysmenorrhea [7].

The pain of dysmenorrhea is caused by uterine hyper-contraction. Calcium is one of the most important manipulators of this contraction [8]. Contractions occur in the smooth muscle upon calcium influx through the calcium channel, where calcium and calmodulin form a complex which activates myosin light chain kinase (MLKL) to induce the extracellular signal-regulated kinase (ERK)/p38 signaling pathway. Furthermore, phosphorylated myosin light chain 20 (MLC-20) is combined with actin, resulting in cross-bridge cycling and uterine contraction [9].

Aromatherapy has been used as a form of pain-relief therapy [10], using essential oils, such as clary sage, marjoram, and lavender oils. Sclareol is one of the compounds found in *Salvia sclarea* essential oil, isolated from *Salvia sclarea* flowers or leaves, and it is classified as a bicyclic diterpene alcohol [11]. It has been noted that sclareol displays antitumor, anti-inflammation, antioxidant, and immune-regulation activity both in vivo and in vitro [12,13,14,15]. The condition of chronic inflammation is highly correlated with pain [16], also indicating that chronic inflammation is related to uterine pain behavior in an endometriosis model [17]. Several natural compounds show high antioxidant and anti-inflammation effects, such as extra virgin olive oil (EVOO) [18,19,20] and resveratrol [21], which can decrease inflammatory cytokine levels and prevent oxidative-related cell death. Sclareol can regulate inflammation by affecting the protein expression of COX-2 [12,13], as well as antioxidant enzyme activity. These reasons indicate that sclareol has the potential to improve dysmenorrhea. In this study, we investigated the anti-dysmenorrhea activates of sclareol in ex vivo and in vivo dysmenorrhea models, as well as its possible mechanism.

## 2. Materials and Methods

### 2.1. Reagent Preparation

Sclareol (CAS: 515-03-7, 98%), oxytocin, sodium bicarbonate, carbachol, acetylcholine (Ach), mannitol, glucose, potassium chloride, potassium phosphate, magnesium sulfate, calcium chloride, estradiol, and dimethyl sulfoxide (DMSO) were purchased from (Sigma-Aldrich, St. Louis, MO, USA). PGF_2α_ and Bay K 8644 were purchased from (Cayman Chemical Company, Ann Arbor, MI, USA).

### 2.2. Ultra-Performance Liquid Chromatography–Mass Spectrometry (UPLC–MS) Analysis of Total Sclareol Content of Salvia sclarea Essential Oil

The *Salvia sclarea* L. essential oil (Can June International Inc., Taipei, Taiwan) was diluted to an appropriate concentration with ethanol and filtrated using a nylon filter (0.22 µm) before being injected into the ultra-performance liquid chromatography–mass spectrometry (UPLC–MS) system. Standard stock solutions of sclareol standard (Sigma-aldrich, St. Louis, MO, USA) were dissolved in ethanol and the calibration range was 20–150 ng/mL with a correlation coefficient of 0.999. The system included a Waters Acquity UPLC equipped with a pump, column compartment, autosampler, and Waters TQS mass spectrometer (Waters, Milford, MA, USA) operated in positive electrospray ion (ESI^+^) mode. The Acquity UPLC HSS T3 (1.8 μm; 2.1 mm × 100 mm) column was employed and maintained at 35 °C with a flow rate at 0.3 mL/min and injection of 2 μL. The mobile phase consisted of 10% (A) and 90% methanol (B), both containing 5 mM ammonium acetate and 0.1% formic acid. The linear gradient conditions were as follows: 1–99% A (0–10 min), 99% B (10–12 min). The ESI parameters were set as follows: capillary voltage, 3.0 kV; cone voltage, 15 V; desolvation temperature, 400 °C; source temperature, 150 °C; desolvation gas flow, 850 L/h; cone gas flow, 150 L/h; nebulizer gas flow, 7.0 bar. Selected ion recording (SIR) mode was used to monitor sclareol at *m*/*z* 331 [M + Na] ^+^. All data were collected using MassLynx 4.1 software spectrometer (Waters, Milford, MA, USA).

### 2.3. Cell Culture

Human uterine smooth muscle cells were obtained from promo cells and were cultured in Dulbecco’s modified Eagle’s medium, nutrient Mixture F-12 (DMEM F12) (Caisson, Taichung City, Taiwan) supplemented with 10% fetal bovine serum (FBS) (Gibco, Grand Island, NY, USA) and 100× penicillin–streptomycin solution (Corning, Manassas, VA, USA) at 37 °C in a humidified 5% CO_2_ incubator.

### 2.4. Reactive Oxygen Species (ROS) Measurement

For treatments, cells were cultured in 96-well plates (1000 cells/well), treated with 1 μM PGF_2α_ and sclareol for 30 min, and incubated with 25 μM 2′,7′-dichlorofluorescin diacetate (DCFDA) for 30 min. A fluorescence microscope was used to capture the fluorescence. We used ImageJ to quantify the ROS density.

### 2.5. Animals

Female imprinting control region (ICR) mice (18–22 g) and Sprague Dawley (SD) rats (200–300 g) were housed in a temperature-controlled and 12 h/12 h artificial illumination room, given ab libitum access to food and water. All animal studies were conducted according to the protocols approved by the IACUC of Taipei Medical University (Permit No. LAC-2019-0350, LAC-2019-0351 and LAC-2019-0645).

### 2.6. Uterine Tissue Preparations and Measurement of Uterine Contraction Ex Vivo

Preparation and measurement were carried out according to previously studies [21,22]. SD rats were sacrificed by CO_2_ and the uterus was surgically removed. Uterine tissue was placed in a container that contained Krebs solution (113 mM NaCl, 4.8 mM KCl, 2.5 mM CaCl_2_, 18 mM NaHCO_3_, 1.2 mM KH_2_PO_4_, 1.2 mM MgSO_4_, 5.5 mM glucose, and 30 mM mannitol; pH 7.4), adherent fat, and connective tissue guardedly removed from the uterus. Each piece of uterine tissue was cut at the same length and set in an isolated organ bath which contained Krebs solution, bubbled with 95% O_2_ and 5% CO_2_ at 37 °C. We preloaded 1 g and equilibrated after at least 30 min. After equilibration, uterine tissue contractions were stimulated using different drugs (10^−6^ M PGF_2α_, 10^−6^ M oxytocin, 10^−5^ M carbachol, 10^−6^ M Ach, 50 mM KCl, and 10^−6^ M Bay K 8644). Sclareol (10, 25, 50, 75 and 100 μM), *Salvia sclarea* essential oil (10, 25, 50, 75, and 100 ppm), or DMSO (solvent control) was added to the organ bath at 10 min intervals. The contraction tension was detecting by force displacement transducers and recorded using LabScribe. To revise for variations between individual uterine contractions, the mean amplitude vales of the pretreatment intervals were expressed as a percentage of control values and taken as 100%.

### 2.7. Measurement of Uterine Contraction Pressure in the In Vivo Study

The method for the measurement of uterine contraction in vivo was modified from previous studies [21,22]. SD rats were anesthetized. We then made a ventral incision at the abdomen and uterus. We put a thin catheter into the uterus, and the catheter was connected to a transducer to detect to pressure transformations, which were recorded using LabScribe software. The rat was injected with oxytocin (1 IU) intraperitoneally (ip) to stimulate uterine hypercontraction, with an ip injection of sclareol (5, 10, or 15 mg/kg) at 20 min intervals. The mean amplitude vales of the pretreatment intervals were expressed as a percentage of control values and taken as 100%.

### 2.8. Measurement of Uterine Tissue under Ca^2+^-Dependent Contractions

This study method was modified from [23]. The uterine tissue was placed in an organ bath containing Ca^2+^-free Krebs solution. After equilibration, sclareol (100 μM) was added; then, the Ca^2+^-free Krebs solution was supplied with increasing concentrations of Ca^2+^, from 0.5 to 5 mM, to restore spontaneous contraction. In another study, the uterine tissues were placed in an organ bath containing Ca^2+^-free Krebs solution. After equilibration, oxytocin (10^−6^ M) was added to the organ bath to induce intracellular Ca^2+^ release from the sarcoplasmic reticulum to produce contractions; then, sclareol (10, 25, 50, 75, and 100 μM) or DMSO (solvent control) was added to the organ bath at 10 min intervals.

### 2.9. Acetic Acid-Induced Writhing Test

Forty-eight female ICR mice were randomly separated into six groups (control, model control, 50 mg/kg sclareol, 100 mg/kg sclareol, and 150 mg/kg sclareol, *n* = 8/group) and were orally pretreated with PBS (control and model control group) and sclareol (50, 100, or 150 mg/kg) 15 min before induction. After pretreatment, all animals received an ip injection of PBS (only control group) or acetic acid (0.6%, 10 mL/kg). The number of writhes was recorded in the 30 min after the ip injection. Analgesia (%) = (model control writhing times − (sclareol writhing times))/model control writhing times.

### 2.10. Oxytocin-Induced Writhing Test

The oxytocin-induced writhing test was carried out according to previous studies [24,25,26]. Forty-eight female ICR mice were randomly separated into six groups (*n* = 4–8/group): control, model control, 50 mg/kg sclareol, 100 mg/kg sclareol, and 150 mg/kg sclareol. All mice were pretreated with estradiol (E_2_, ip, 1 mg/kg) for seven consecutive days. Different doses of sclareol (50, 100, or 150 mg/kg) were administered orally for three days before ip injection E_2_. On day 7, oxytocin (70 IU/kg) was given as an ip injection after pretreatment for 15 min. The number of writhes was recorded in the 30 min after the ip injection. After the test, mice were sacrificed, and uterine tissue was collected for further analysis. Analgesia (%) = (model control writhing times − (sclareol writhing times))/model control writhing times.

### 2.11. Lipid Peroxidation Determination

The lipid peroxidation was measured using the level of malondialdehyde (MDA) in the serum. We used a 2-thiobarbituric acid-reacting substances test (TBARS) assay kit and followed the manufacturer’s instructions. Results were measured on a 532 nm plate using a VERSA Max microplate reader (Molecular Devices, San Jose, CA, USA).

### 2.12. Western Blotting Analysis

Tissue proteins were homogenized by means of a radioimmunoprecipitation assay buffer containing protease inhibitor and phosphatase inhibitor (Roche Mannheim, Baden-Württemberg, Germany). Protein samples were quantitated using the bicinchoninic acid assay (BCA) (T-Pro Biotechnology, Dublin, UK). In total, 40–60 μg of protein in the sample was separated by 10–12% SDS-PAGE. The protein was transferred to 0.22 µm poly (vinylidene fluoride) (PVDF) membranes and blocked with 5% bovine serum albumin (BSA) for 1 h at room temperature (RT). The membrane was incubated with primary antibodies, including oxytocin receptor (OTR) (1:500, sc-8103, Santa Cruz), myosin light-chain kinase (MLCK) (1:1000, sc-25428, Santa Cruz), cyclooxygenase-2 (COX-2) (1:200, 160126, Cayman), phosphorylated extracellular signal-regulated kinase (p-ERK) (1:1000, #9101, Cell signaling), ERK (1:1000, #9102, Cell signaling), p-p38 (1:1000, #9211, Cell signaling), p38 (1:1000, #9212, Cell signaling), phosphorylated myosin light chain-20 (p-MLC20) (1:1000, #3675, Cell signaling), MLC20 (1:1000, sc-28319, Santa Cruz), and α-actin (1:5000, sc-32251, Santa Cruz) overnight at 4 °C. Then, the membranes were incubated with a secondary horseradish peroxidase (HRP) -conjugated antibody (anti-mouse or anti-rabbit, 1:10000 or anti-goat, 1:500, RT, 2 h) and visualized using enhanced chemiluminescence (ECL) (T-Pro Biotechnology). Results were quantitated using ImageJ software.

### 2.13. Statistical Analysis

All results are expressed as the means ± standard error of the mean (SEM), analyzed using GraphPad Prism version 5.0 software (GraphPad, San Diego, CA, USA). Calculation of statistically significant differences was performed using one-way analysis of variance (ANOVA) with Tukey’s post hoc test or the Mann–Whitney U test. A Student’s unpaired *t*-test was used for comparison between two groups. Significance was accepted at *p* < 0.05.

## 3. Results

### 3.1. Effect of Salvia sclarea L. Essential Oil on PGF_2α_-Induced Uterine Contractions

**Figure 1:**
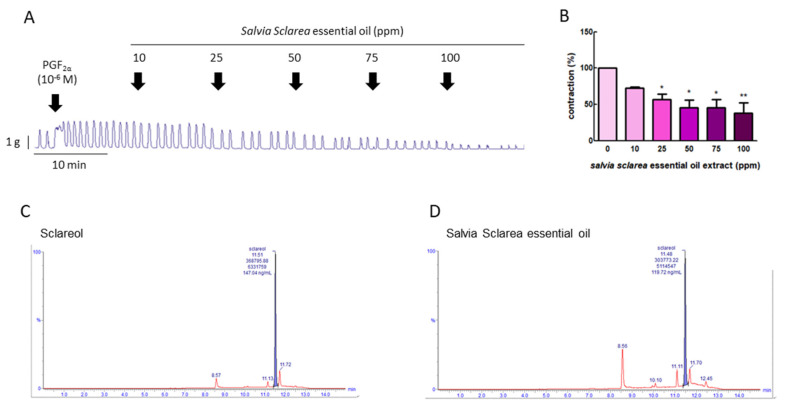
Effect of *Salvia sclarea* L. essential oil on prostaglandin (PG) F_2α_-induced uterine contraction and total sclareol content in *Salvia sclarea* L. essential oil. (**A**) Representative recordings of rat uterine tissue were induced with PGF_2α_ (10^−6^ M) along with exposure of rat uterine smooth muscles to essential oil (at 10, 25, 50, 75, and 100 ppm.) (**B**) Dose-dependent effects of essential oil on the mean peak amplitude. Each column represents the mean ± standard error of the mean (SEM); *n* = 4; * *p* < 0.05, ** *p* < 0.001 compared to 0. (**C**) Ultra-performance liquid chromatography (UPLC)–MS/MS chromatogram of sclareol standard. (**D**) UPLC–MS/MS chromatogram of *Salvia Sclarea* essential oil.

To explore whether *Salvia sclarea* L. essential oil had the potential to inhibition uterine contraction, we first used PGF_2α_ to induce uterine contraction, as PGF_2α_ is the major factor leading to dysmenorrhea. Figure 1A,B show that PGF_2α_ (10^−6^ M) significantly increased the contraction amplitude, and, when exposed to *Salvia sclarea* essential oil (at 10, 25, 50, 75, and 100 ppm), the contraction amplitude was inhibited in a dose-dependent manner; at 25–100 ppm, it was significantly inhibited. This verifies that *Salvia sclarea* essential oil has the potential to inhibit uterine contraction.

### 3.2. Total Sclareol Content of Salvia sclarea L. Essential Oil

To evaluate the effective compound in *Salvia sclarea* L. essential oil, the total sclareol content of *Salvia sclarea* essential oil was analyzed using UPLC-MS/MS. We found that the retention time of sclareol was 11.51 min, and that of clary sage oil was 11.48 min. The sclareol content in clary sage oil was 0.239%, according to the calibration curve (Figure 1C,D).

### 3.3. Effect of Sclareol on Oxytocin-Induced Uterine Contraction In Vivo

**Figure 2:**
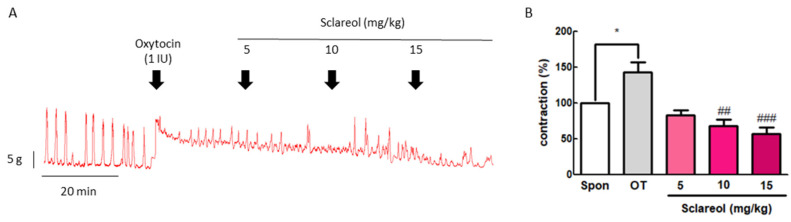
Effect of sclareol on oxytocin-induced uterine contraction in vivo and its possible mechanism. (**A**) Effect of sclareol (5, 10, or 15 mg/kg) on oxytocin-induced (1 IU) uterine contractions in vivo. The rats were ip injected with oxytocin combined with sclareol (5, 10, or 15 mg/kg), and the contractions were recorded. (**B**) The effects of sclareol on the mean peak amplitude. * *p* < 0.05 compared to spontaneous contraction (Spon), ^##^ *p* < 0.01 compared to oxytocin, ^###^ *p* < 0.001 compared to oxytocin. Each column represents the mean ± SEM; *n* = 7.

To confirm the inhibitive effect of sclareol in vivo, we examined the uterine contraction pressure of rats. The rats were ip injected with oxytocin (1 IU) to induce uterine contraction, and then ip injected with 5, 10, or 15 mg/kg sclareol. The intervention of sclareol was able to significantly reduce the uterine contraction pressure, proving that sclareol is able to affect the uterine contractions in vivo (Figure 2).

### 3.4. Effect of Sclareol on PGF_2α_-, Oxytocin-, Acetylcholine-, and Carbachol-Induced Uterine Contractions

**Figure 3:**
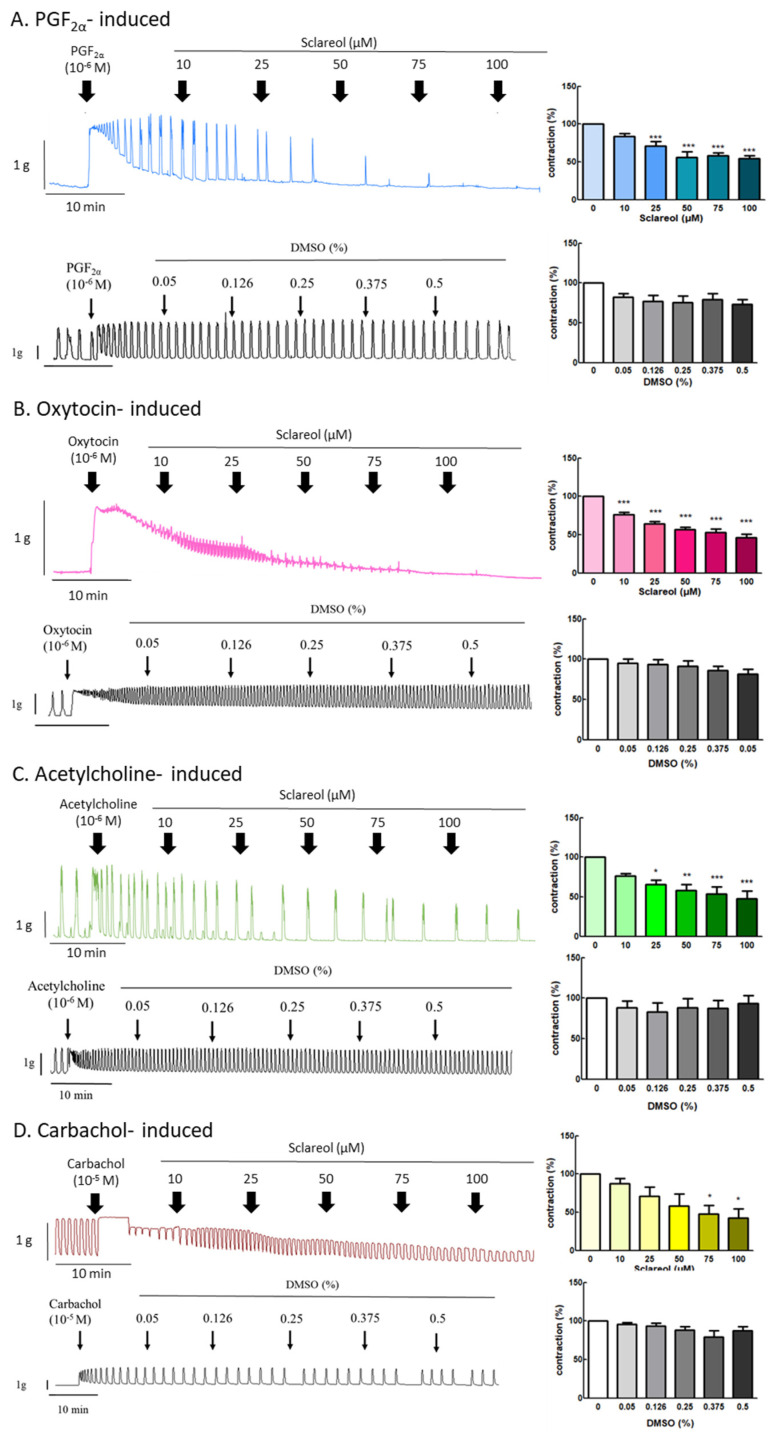
Effect of sclareol on drug-induced uterine contraction. Representative recordings of rat uterine tissues were induced with (**A**) 10^−6^ M PGF2α, (**B**) 10^−6^ M oxytocin, (**C**) 10^−5^ M carbachol, and (**D**) 10^−6^ M acetylcholine Ach, with exposure of rat uterine smooth muscles to sclareol (10, 25, 50, 75, and 100 μM). Dose-dependent effects of sclareol on the mean peak amplitude. Each column represents the mean ± SEM; *n* = 4–8. * *p* < 0.05, ** *p* < 0.01 and *** *p* < 0.001 compared to 0 (drug-induced only).

Since sclareol is contained in *Salvia sclarea* L. essential oil, we further examined the effect of sclareol on uterine contractions, to investigate whether sclareol can affect uterine contractions via different pathways. We, thus, examined the effect of sclareol on PGF_2α_-, oxytocin-, acetylcholine-, and carbachol-induced uterine contractions. PGF_2α_ is the main reason for the increase in dysmenorrhea. Oxytocin, acetylcholine, and carbachol are intended to release intracellular stored Ca^2+^. As a result, PGF_2α_ (10^−6^ M) can significantly increase the contraction amplitude, and, at concentrations of 25–100 μM, sclareol was significantly inhibited, showing that sclareol can possibly be used to treat uterine hypercontraction (Figure 3A). Sclareol significantly decreased oxytocin-, acetylcholine-, and carbachol-induced uterine contraction in different dosages (Figure 3B,D). The vehicle (DMSO) did not affect oxytocin-, acetylcholine-, and carbachol-induced uterine contraction.

### 3.5. Effect of Sclareol on KCl- and Bay K 8644-Induced Uterine Contractions

**Figure 4:**
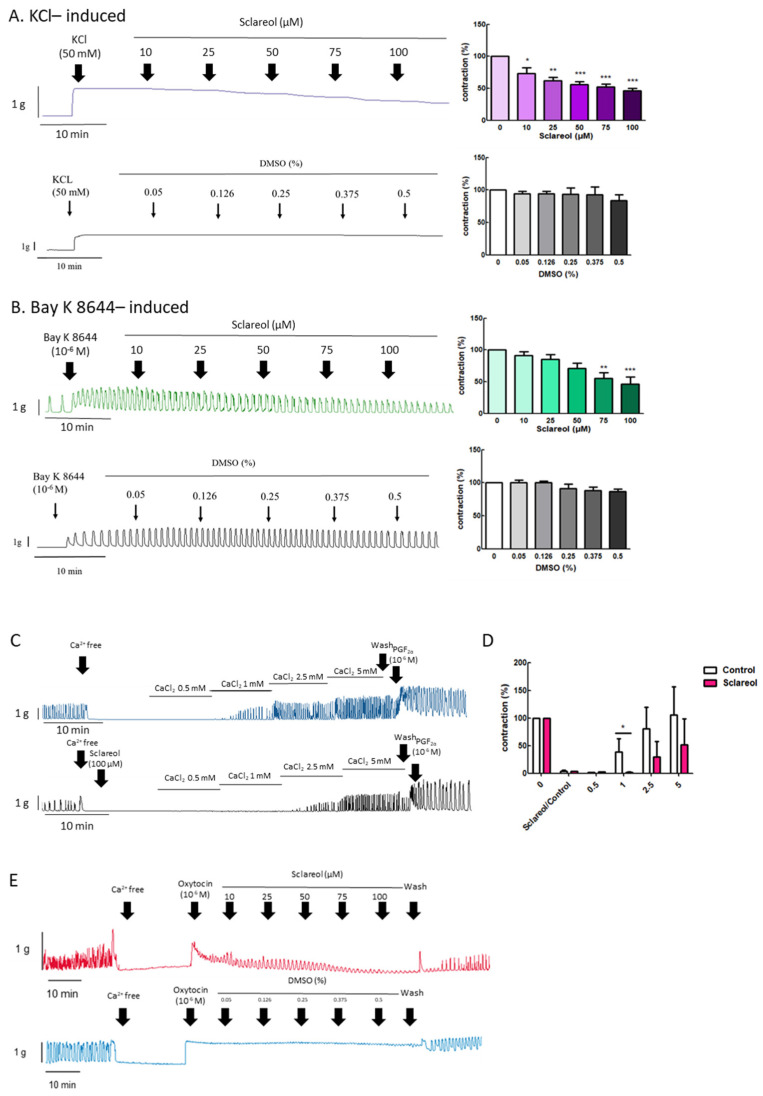
Effect of sclareol on Ca^2+^-dependent contractile responses. Representative recordings of rat uterine contractions were induced with (**A**) 50 mM KCl and (**B**) 10^−6^ M Bay K 8644, with exposure of rat uterine smooth muscles to sclareol (10, 25, 50, 75, and 100 μM). Dose-dependent effects of sclareol on the mean peak amplitude. Each column represents the mean ± SEM; *n* = 4–8. * *p* < 0.05, ** *p* < 0.01 and *** *p* < 0.001 compared to 0 (drug-induced only). Inhibitory actions of sclareol on Ca^2+^-dependent contractile responses. (**C**) Muscle segments were initially pretreated in a Ca^2+^-free medium containing calcium (0.5–5 mM) only or plus sclareol (100 μM), and calcium was then cumulatively applied to trigger muscle contraction. (**D**) Inhibitive effect of sclareol on the mean peak amplitude. * *p* < 0.05 compared to control; *n* = 3. (**E**) Rat uterine contractions were induced with oxytocin (OT) (10^−6^ M) and exposure to sclareol (10, 25, 50, 75, and 100 μM) or DMSO (0.05%, 0.126%, 0.25%, 0.375%, and 0.5%).

To explore the effect of extracellular calcium on uterine contraction, we used KCl to create High-K^+^ conditions due to membrane depolarization, causing extracellular Ca^2+^ entry through the voltage-dependent calcium channel. Bay K 8644 is an L-type Ca^2+^-channel activator that increases the entry of extracellular Ca^2+^. Administration of sclareol along with KCl (10–100 μM) or Bay K 8644 (75–100 μM) resulted in a dose-dependent decrease in uterine contractions (Figure 4A,B).

### 3.6. Effect of Sclareol on Ca^2+^-Dependent Contractions

When uterine tissue is in Ca^2+^ free conditions, the spontaneous contractions were invalidated. Along with an increase in Ca^2+^ concentrations, the spontaneous contractions were recovered. To test the calcium-dependent contraction, we used a calcium-free solution for further examination. When in the Ca^2+^-free Krebs solution combined with sclareol treatment, the contractions declined (Figure 4C,D). As shown in Figure 4E, oxytocin (10^−6^ M) was added to an organ bath to increase the calcium influx, which recovered the uterine contraction, and sclareol showed the inhibition of calcium influx-induced contraction. This demonstrates that sclareol can effectively decrease calcium-dependent contractions.

### 3.7. Effect of Sclareol on PGF_2α_-Induced Uterine Contraction-Related Protein Expression

**Figure 5:**
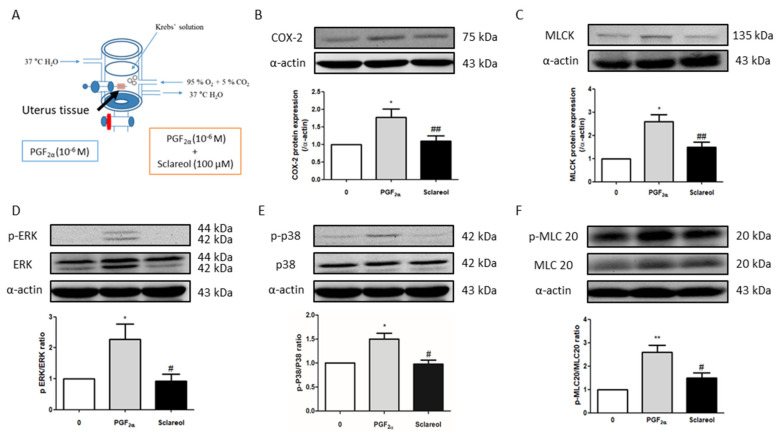
Possible mechanism underlying the effect of sclareol on PGF_2α_-induced uterine contraction. (**A**) Tissue collection method. After PGF_2α_-induced uterine contraction in the presence of 0 and 100 μM sclareol, the uterus was taken out (at 30 min). (**B**–**F**) Protein expression of cyclooxygenase-2 (COX-2), myosin light-chain kinase (MLCK), phosphorylated extracellular signal-regulated kinase (p-ERK), ERK, p-p38, p38, phosphorylated myosin light chain 20 (p-MLC20), MLC20, and α-actin during PGF_2α_-induced contraction at 30 min in rat uterus; *n* = 3–6; * *p* < 0.05, ** *p* < 0.001 compared to 0; ^#^ *p* < 0.05, ^##^ *p* < 0.01 compared to PGF_2α_. Each column represents the mean ± SEM.

To examine the possible mechanism of sclareol’s inhibition of uterine contractions, we used Western blot analysis to observe the uterine contraction-related protein expression. Uterine tissue was collected after treatment with PGF_2α_ or in combination with sclareol (100 μM) for 30 min. Uterine tissue that was treated with the sclareol combination presented significantly lower protein expression of COX-2, MLCK, p-ERK, p-p38, and p-MLC20 compared with the uterine tissue treated with PGF_2α_, illustrating that sclareol inhibited uterine contraction by affecting calcium-related signaling and protein expression (Figure 5B,F).

### 3.8. Effect of Sclareol on Acetic Acid-Induced Writhing Test

To evaluate analgesic effects, we used the acetic acid-induced writhing model. The acetic acid solution significantly increased writhing times in the model control group and attained 62.5 times more writhing in 30 min. On the other hand, intervention with sclareol (at 50, 100, and 150 mg/kg) was able to significantly reduce the writhing times compared to the model control group, demonstrating that sclareol possesses analgesic effects (Table 1).

### 3.9. Effect of Sclareol on Oxytocin-Induced Writhing Test

To further evaluate the inhibition of uterine hypercontraction pain, we used an oxytocin-induced writhing model. The injection of oxytocin could significantly increase writhing times in model control groups, and the administration of sclareol (at 50, 100, and 150 mg/kg) could significantly decrease the writhing times in 30 min. This demonstrates that sclareol has the potential to improve dysmenorrhea (Table 1).

### 3.10. Effect of Sclareol on Oxidative Stress in Uterine Smooth Muscle Cell and Dysmenorrhea Mice

**Figure 6:**
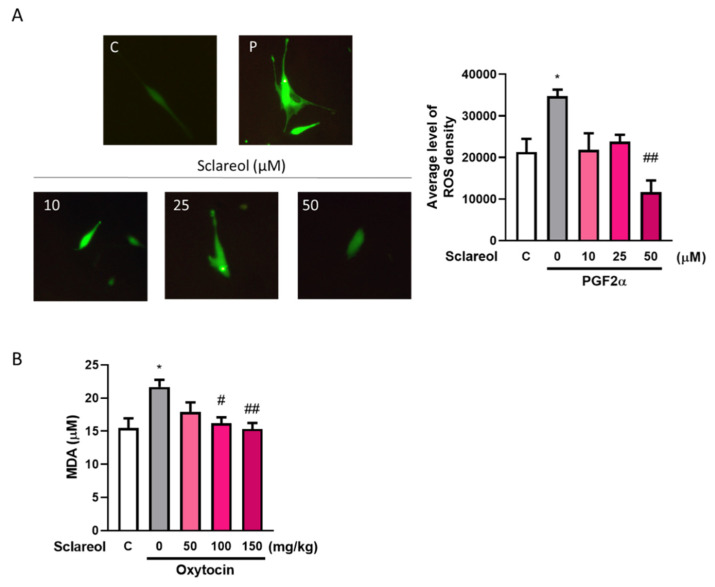
Effect of sclareol on oxidative stress in uterine smooth muscle cell and dysmenorrhea mice. (**A**) Reactive oxygen species (ROS) density in in vitro study. * *p* < 0.05 compared with control group, ^##^ *p* < 0.01 compared with sclareol 0 μM group; C, control group. (**B**) Serum MDA concentration in oxytocin-induced writhing test. * *p* < 0.05 compared with control group; ^#^ *p* < 0.05, ^##^ *p* < 0.01 compared with sclareol 0 mg/kg group; *n* = 3–8; the graph was captured at 20× magnification.

A significant increase in the serum MDA level was observed in the oxytocin-treated group compared with the control group. As treatment with sclareol decreased significantly, oxytocin-induced oxidative stress increased. We used an in vitro method to examine the ROS fluorescence density. The results showed that the PGF2α-induced group had a significantly higher ROS density, and a high dose of sclareol could eliminate the increase in ROS density. This shows that the high antioxidant ability of sclareol plays an important role in the inhibition of dysmenorrhea (Figure 6).

### 3.11. Effect of Sclareol on Oxytocin-Induced Uterine Contraction-Related Protein Expression

**Figure 7:**
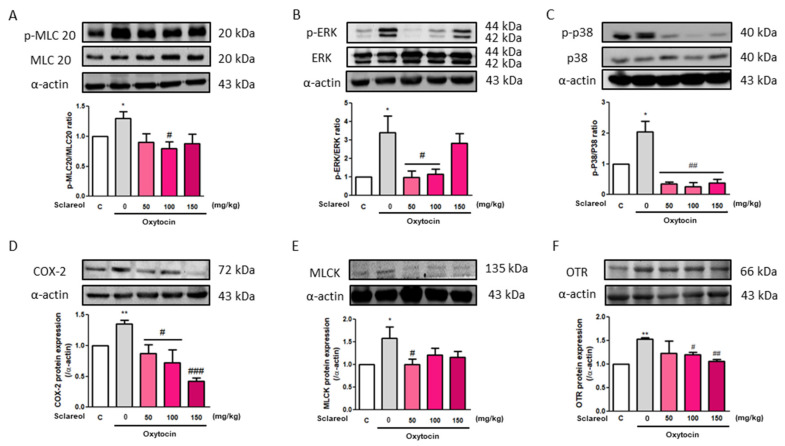
Effect of sclareol on uterine contraction-related protein expression in oxytocin-induced writhing test model. The (**A**) p-MLC20/MLC20, (**B**) p-ERK/ERK, (**C**) p-p38/p38, (**D**) COX-2, (**E**) MLCK, and (**F**) oxytocin receptor (OTR) levels were detected by Western blot. C, Control; 0, model control group; 50, sclareol 50 mg/kg; 100, sclareol 100 mg/kg; 150, sclareol 150 mg/kg; *n* = 4–6; * *p* < 0.05, ** *p* < 0.01 compared to C; ^#^ *p* < 0.05, ^##^ *p* < 0.01, ^###^ *p* < 0.001 compared to the sclareol 0 mg/kg group. Each column represents the mean ± SEM; *n* = 3–5.

To investigate the possible mechanism underlying sclareol’s improvement of writhing times in the oxytocin-induced writhing test, we used Western blot analysis to observe the uterine contraction-related protein expression. We found that the intervention of sclareol resulted in a lower protein expression of p-MLC20/MLC20, p-ERK/ERK, p-p38/p38, COX-2, MLCK, and OTR. These results, as well as those of the earlier Western blot study from rat uterine tissue, prove that sclareol improves dysmenorrhea by affecting calcium-related signaling and protein expression (Figure 7).

## 4. Discussion

Sclareol is a labdane-type diterpenes compound, which was first extracted from the plant *Salvia sclarea* L. (Lamiaceae). Sclareols are widely used in the food and cosmetic industries and as supplements. Recently, it has been widely used in anticancer [27] and anti-inflammation experiments [12]. Sclareol inhibited lipopolysaccharide-induced lung injury via the inhibition of p-ERK and p-p38 protein expression to reduce the mitogen-activated protein kinase (MAPK) signaling transduction pathway, further inhibiting COX-2 expression and having an antioxidant effect, decreasing the number of reactive oxygen species (ROS) and inflammation [12]. Furthermore, in cancer research, it was shown that sclareol has an outstanding effect on the inhibition of the ERK-related signaling pathway [28]. These effects of sclareol demonstrate the capacity for *Salvia sclarea* essential oil to be used for the improvement of dysmenorrhea symptoms [29]. It has been used as an abdominal massage oil, and it was proven to be effective in decreasing the severity of dysmenorrhea [30]. The results of the present study demonstrate that *Salvia sclarea* essential oil reduced uterine contractions in a PGF_2α_-induced rat uterine contraction model, illustrating that *Salvia sclarea* essential oil can improve dysmenorrhea via the reduction of uterine hypercontraction.

In this study, we used different agents to induce uterine contraction, and those agents acted on different receptors, such as PGF_2α_ acting on the FP receptor, oxytocin acting on OTR [31], acetylcholine and carbachol acting on the muscarinic acetylcholine receptor (M3) receptor [32], and Bay K 8644 and KCl affecting the voltage-gated calcium channels (VDCC) [33,34]. Although uterotonic hormones (PGF_2α_, oxytocin, acetylcholine, and carbachol) act on different receptors, they all induce uterine contraction via increases in extracellular Ca^2+^ entry and intracellular Ca^2+^ release. Moreover, Bay K 8644 (a Ca^2+^ channel activator) and KCl solution (which creates high K^+^ conditions, leading to membrane depolarization) increase extracellular Ca^2+^ via the VDCC [21]. In addition, when the uterine tissue is in Ca^2+^-free Krebs solution, oxytocin can induce contraction via intracellular Ca^2+^ release from the sarcoplasmic reticulum [23]. Obviously, sclareol showed inhibitory effects in this study, demonstrating that the relaxation effect induced by sclareol can take place via several receptors, thus affecting Ca^2+^ level.

Phosphorylated MLC20 (p-MLC20) plays a pivotal role in regulating uterine smooth muscle contraction, and it is the only known physiological substrate of MLCK [35]. Phosphorylated ERK is one of the proteins that can active MLCK [25,36]. p-ERK also acts as a signaling trigger in several pathways. Previous studies showed that sclareol can significantly decrease the activity of the p38-AMPK signaling pathway [37]. In cancer research, sclareol not only showed an anticancer effect, but also had the ability to target the mitogen-activated protein kinase (MAPK)/extracellular signal-regulated kinase (ERK) signaling pathway [28], showing that sclareol can modulate the ERK/p38 signaling pathway. This is the first study to claim the effect of sclareol on the calcium-related muscle contraction signaling pathway. Our results showed that p-ERK, MLCK, and p-MLC20 protein expression was increased in PGF_2α_-induced rat uterine tissue, and that intervention with sclareol was able to reduce the expression of those proteins, indicating that the effect of sclareol on uterine contraction may take place by affecting p-ERK, MLCK, and p-MLC20 protein expression.

PGF_2α_ is a critical factor in dysmenorrhea and a product of COX-2. Hence, nonsteroidal anti-inflammatory drugs (NSAIDs) have been used as a first-line therapy for treating dysmenorrhea. NSAIDs are a cyclooxygenase inhibitor; they can inhibit COX, reducing prostaglandin production. However, NSAIDs have around a 30% failure rate [38], and disorders of the liver, kidney, and digestive system can occur when using them in the long term. In a previous study, it was shown that p-p38 is a protein that can regulate Phospholipase A2 (PLA_2_) activity, decreasing the production of arachidonic acid and PGH_2_ [39], which is the first intermediate in the biosynthesis of all PGs [6]. In our study, we found that p-p38 protein expression was increased in PGF_2α_-induced rat uterine tissue, suggesting that sclareol potently improves inflammatory conditions in dysmenorrhea by affecting p-p38 protein expression.

The acetic acid-induced writhing test has been used as a screening tool for peripheral analgesic or anti-inflammatory new substances [40]. When mice are ip injected with acetic acid solutions, proinflammatory substances are released, activating peripheral nociceptors, leading to pain and writhing responses [24]. Compared to the control group, a significant reduction in writhing times is considered an antinociceptic response [41]. Sclareol produced significant reductions in writhing times, suggesting that sclareol exerts a peripheral analgesic effect. Although it did not show a dose-dependent result, this can be explained by the suggestion that a low dose of sclareol exerted central control at the brain or spinal cord level to provide an analgesic effect [42]. A high dose of sclareol can provide an anti-inflammatory effect; however, it takes more than 15 min to act.

To further explore the possibility of using sclareol to relieve dysmenorrhea, an oxytocin-induced writhing test was carried out. The oxytocin-induced writhing test is a reliable model for primary dysmenorrhea [43]. This model reflects the clinical features and pathogenesis of dysmenorrhea, such as uterine contraction, PG synthesis, pain, and Ca^2+^ entry [26]. Pretreatment with estrogen could increase the oxytocin receptor (OTR) protein expression and increase the writhing responses induced by oxytocin [44]. In this study, oxytocin significantly increased writhing times and OTR protein expression, as in previous studies, and the intervention with sclareol attenuated writhing times and OTR protein expression. Equally, sclareol inhibited oxytocin-induced uterine contraction ex vivo. These findings imply that sclareol can reduce dysmenorrhea.

## 5. Conclusions

**Figure 8:**
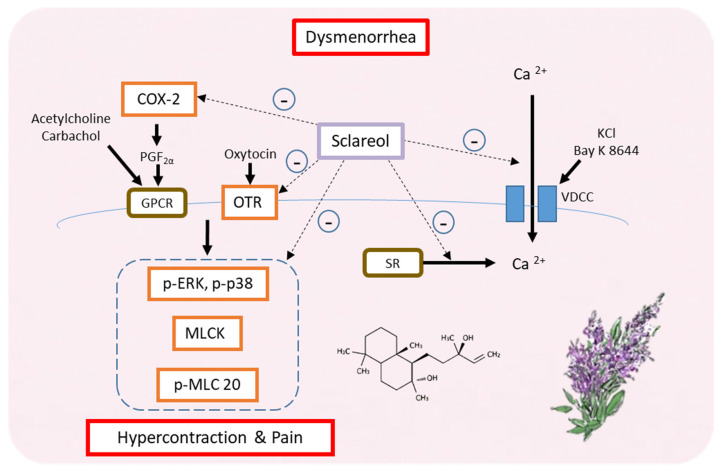
Possible model for the regulation of dysmenorrhea by sclareol in ex vivo and in vivo dysmenorrhea models. In summary, sclareol can downregulate the protein expression of OTR, MLCK, COX-2, p-ERK, p-p38, and p-MLC20 and can regulate the concentration of intracellular calcium, thus having the potential to regulate dysmenorrhea.

Our findings suggest that sclareol is a potential natural product that can treat primary dysmenorrhea. In fact, this is the first study showing the relationship between sclareol and dysmenorrhea. Furthermore, sclareol improved dysmenorrhea via downregulating the protein expression of OTR, MLCK, COX-2, p-ERK, p-p38, and p-MLC20 and regulating the concentration of intracellular calcium. Hence, sclareol may ameliorate primary dysmenorrhea and inflammation (Figure 8).

## Figures and Tables

**Table 1:** Effect of sclareol on acetic acid- and oxytocin-induced writhing tests.

Model		Acetic Acid- Induced		Oxytocin-Induced(Pretreat Sclareol)	
Group		Writhing Times /30 min	Analgesia (%)	Writhing Times /30 min	Analgesia (%)
Control		0.0 ± 0.0	-	0.0 ± 0.0	-
Model control (MC)		62.5 ± 18.6 ***	-	17.4 ± 5.6 ***	-
Sclareol (mg/kg)	50	24.6 ± 16.5 ^##^	60.7	4.0 ± 3.3 ^###^	77
	100	30.4 ± 11.0 ^##^	51.3	3.5 ± 2.8 ^###^	79.9
	150	36.2 ± 19.8 ^#^	42.1	1.8 ± 1.7 ^###^	89.9

Analgesia (%) = (Model control writhing times—(sclareol writhing times))/model control writhing times. *** *p* < 0.001 compared to control group, ^#^ *p* < 0.05 compared to model control group, ^##^ *p* < 0.01 compared to model group, ^###^ *p* < 0.001 compared to model group. Each column represents the mean ± SEM; *n* = 4–8.
